# Current management and future perspectives of covert hepatic encephalopathy in Japan: a nationwide survey

**DOI:** 10.1007/s00535-025-02232-0

**Published:** 2025-03-07

**Authors:** Takao Miwa, Mio Tsuruoka, Hajime Ueda, Tamami Abe, Hiroki Inada, Yoshimi Yukawa-Muto, Masatsugu Ohara, Taeang Arai, Yasuyuki Tamai, Hiroshi Isoda, Tomoko Tadokoro, Tatsunori Hanai, Takanori Ito, Nobuharu Tamaki, Akira Sakamaki, Yoshihiko Aoki, Fujimasa Tada, Sachiyo Yoshio, Hirokazu Takahashi, Asahiro Morishita, Tsuyoshi Ishikawa, Jun Inoue, Goki Suda, Chikara Ogawa, Masanori Atsukawa, Atsushi Hiraoka, Hidekatsu Kuroda, Tadashi Namisaki, Takashi Honda, Takumi Kawaguchi, Yasuhito Tanaka, Shuji Terai, Tadashi Ikegami, Hitoshi Yoshiji, Motoh Iwasa, Masahito Shimizu

**Affiliations:** 1 Department of Gastroenterology/Internal Medicine, Graduate School of Medicine, Gifu University, 1-1 Yanagido, Gifu, 501-1194 Japan; 2 Division of Gastroenterology, Tohoku University Graduate School of Medicine, 1-1 Seiryo-Machi, Aoba-Ku, Sendai, 980-8574 Japan; 3 Division of Gastroenterology and Hepatology, Tokyo Medical University Ibaraki Medical Center, 3-20-1 Chuo, Ami-Machi, Inashiki-Gun, Ibaraki, 300-3095 Japan; 4 Division of Gastroenterology and Hepatology, Department of Internal Medicine, Iwate Medical University, Iwate Medical University School of Medicine, Nishitokuta 2-1-1, Yahaba-Cho, Shiwa-Gun, Yahaba, Iwate 028-3694 Japan; 5 Department of Gastroenterology and Hepatology, Faculty of Life Sciences, Kumamoto University, Kumamoto University, 1-1-1, Honjo, Chuo-Ku, Kumamoto, 860-8556 Japan; 6 Department of Hepatology, Graduate School of Medicine, Osaka Metropolitan University, 1-4-3 Asahimachi, Abeno-Ku, Osaka, 545-8585 Japan; 7 Department of Gastroenterology and Hepatology, Hokkaido University Graduate School of Medicine, Kita-15 Nishi-7, Kita-Ku, Sapporo-Shi, Hokkaido, 060-8638 Japan; 8 Division of Gastroenterology and Hepatology, Nippon Medical School, 1-1-5 Sendagi, Bunkyo-Ku, Tokyo, 113-8603 Japan; 9 Department of Gastroenterology and Hepatology, Mie University Graduate School of Medicine, 2-174 Edobashi, Tsu, 514-8507 Japan; 10 Liver Center, Saga University Hospital, 5-1-1 Nabeshima, Saga, 849-8501 Japan; 11 Department of Gastroenterology and Neurology, Faculty of Medicine, Kagawa University, 1750-1 Ikenobe, Miki, Kita, Takamatsu, Kagawa 761-0793 Japan; 12 Department of Gastroenterology and Hepatology, Nagoya University Graduate School of Medicine, 65 Tsurumai-Cho, Showa, Nagoya 466-8550 Japan; 13 Department of Gastroenterology and Hepatology, Musashino Red Cross Hospital, 1-26-1Kyonan-Cho, Musashino-Shi, Tokyo, 180-8610 Japan; 14 Division of Gastroenterology and Hepatology, Graduate School of Medical and Dental Sciences, Niigata University, 1-757 Asahimachidori, Chuo-Ku, Niigata, 951-8510 Japan; 15 Kohnodai Hospital, National Center for Global Health and Medicine, 1-7-1, Kohnodai, Ichikawa, 272-8516 Japan; 16 Gastroenterology Center, Ehime Prefectural Central Hospital, 83 Kasuga-Cho, Matsuyama, Ehime 790-0024 Japan; 17 Department of Human Immunology and Translational Research, National Center for Global Health and Medicine, 1-21-1, Toyama, Shinjuku-Ku, Tokyo, 162-8655 Japan; 18 Department of Gastroenterology and Hepatology, Yamaguchi University Graduate School of Medicine, 1-1-1 Minami-Kogushi, Ube-Yamaguchi, 7558505 Japan; 19 Department of Gastroenterology and Hepatology, Takamatsu Red Cross Hospital, Takamatsu, 4-1-3 Bancho, Takamatsu City, Kagawa Prefecture 760-0017 Japan; 20 Department of Gastroenterology, Nara Medical University, 840 Shijo-Cho, Kashihara, Nara 634-8521 Japan; 21 Division of Gastroenterology, Department of Medicine, Kurume University School of Medicine, 67 Asahi-Machi, Kurume, 830-0011 Japan

**Keywords:** Animal naming test, Liver cirrhosis, Minimal hepatic encephalopathy, Multidisciplinary team, Stroop test

## Abstract

**Background:**

Covert hepatic encephalopathy (CHE) leads to devastating outcomes in patients with cirrhosis. This study aims to elucidate the current management and future perspectives of CHE in Japan.

**Methods:**

A questionnaire-based cross-sectional study was conducted among physicians involved in managing cirrhosis in Japan. The primary aim was to elucidate the real-world management of CHE, including testing and treatment. Factors influencing the implementation of CHE testing were analyzed using a logistic regression model. Limitations and future perspectives for improving the management of CHE were also evaluated.

**Results:**

Of 511 physicians surveyed, 93.9% recognized CHE as a significant problem, and 86.9% agreed that it should be tested. Overall, 62.8% of physicians tested for CHE, whereas 37.2% did not. Multivariable analysis identified institutional factors and certifying board as significant determinants of CHE test implementation. The Stroop (68.2%) and neuropsychiatric tests (57.5%) were the most commonly used methods of identifying CHE. Among those who tested for CHE, 87.7% treated CHE; the most common treatments were lactulose (81.5%), rifaximin (76.3%), and branched-chain amino acids (70.4%). Among non-testers, the primary barrier was the time requirement for testing. Proposals to encourage CHE testing included the development of simple tests and integration of multidisciplinary teams.

**Conclusions:**

Most physicians involved in cirrhosis care in Japan recognize CHE as a significant problem that warrants testing. However, testing for CHE remains limited by institutional factors and physician specialties. Time requirements for CHE testing are the primary barrier, and simple tests and multidisciplinary teams are recommended to enhance CHE management.

**Supplementary Information:**

The online version contains supplementary material available at 10.1007/s00535-025-02232-0.

## Introduction

Covert hepatic encephalopathy (CHE) is the mildest form of neurocognitive impairment caused by impaired liver functional reserve in patients with cirrhosis [1–5]. Although CHE is asymptomatic, its diagnosis is crucial, as it affects approximately 20–40% of patients with cirrhosis and progresses to overt hepatic encephalopathy (OHE) at an annual incidence rate of 10% [6–8]. In addition, accumulating evidence highlights the significant impact of CHE in reduced quality of life (QOL), a higher incidence of falls and motor vehicle accidents, and increased mortality [1–5, 9]. Therefore, early detection and appropriate management of CHE are important to prevent devastating outcomes in patients with cirrhosis, and several global liver disease societies recommend screening for CHE in patients with cirrhosis [1–5]. However, only a small proportion of patients with cirrhosis are likely to be screened for CHE in real-world clinical practice.

Ideally, all patients with cirrhosis should be screened for CHE [3]. However, physicians involved in cirrhosis care recognize several gaps between the evidence on CHE and real-world practice, which limit the generalization of CHE management. For instance, the extent to which physicians caring for patients with cirrhosis recognize CHE as a significant clinical concern remains unclear. Consequently, the proportion of patients with cirrhosis who undergo CHE testing has yet to be explored. In addition, the decision to test for CHE may be influenced by the clinical context, the time required, the availability of diagnostic tools and standardized tests, and the presence of well-trained practitioners [1–3]. Furthermore, the lack of evidence connecting CHE to clinical outcomes or treatment efficacy may affect clinicians’ decision-making in the management of cirrhosis [3]. Few studies have assessed the current state of CHE management, which could provide valuable guidance for both clinical practice and research [10]. Therefore, we hypothesized that examining real-world CHE management could generate valuable evidence to refine clinical practice and guide future research.

This study aimed to clarify real-world CHE management practices in Japan, including testing and treatment, through a nationwide questionnaire survey. In addition, we investigated the factors influencing the decision to perform CHE testing. Furthermore, we analyzed the limitations of CHE testing and proposed strategies to address these challenges, providing insights to enhance cirrhosis care and guide future research.

## Methods

### Study design and participants

This nationwide questionnaire-based cross-sectional study recruited physicians in cirrhosis care in Japan between August and November 2024. A CHE management questionnaire was distributed through hepatologists affiliated with 20 collaborating institutions across Japan (one from Hokkaido, two from Tohoku, three from Kanto, four from Chubu, two from Kansai, two from Chugoku, three from Shikoku, and three from Kyushu–Okinawa regions). Physicians involved in cirrhosis care at any institution or region in Japan who agreed to participate were included. Exclusion criteria were physicians without knowledge of CHE and those with incomplete baseline characteristics or questionnaire responses. The study’s purpose was explained in the consent form, and all participants provided informed consent. The study protocol was reviewed and approved by the Institutional Review Board of the Gifu University Graduate School of Medicine (approval number: 2024-133). This study adhered to the ethical principles outlined in the 2013 Declaration of Helsinki.

### A questionnaire for CHE management

An anonymized questionnaire on CHE management was administered online via Microsoft Forms (Microsoft Corporation, Redmond, WA, USA). The questionnaire was adapted from a survey conducted among members of the American Association for the Study of Liver Diseases (AASLD) [10]. The questionnaire included nine items: (Q1) Is CHE a significant problem?; (Q2) Should CHE be tested for?; (Q3) How often do you test for CHE?; (Q4) Which tests for CHE are conducted in your practice?; (Q5) Why do you test for CHE?; (Q6) Do you treat CHE?; (Q7) Which medications do you use to treat CHE?; (Q8) Why don’t you test for CHE?; and (Q9) What will increase your likelihood of testing for CHE? The questionnaire was designed to evaluate physicians' perceptions of CHE and the frequency of CHE testing (Q1–Q3). For physicians who performed CHE testing, the survey included questions about the types of tests used, reasons for testing, and details of administered treatments (Q4–Q7). For those who did not test for CHE, the reasons for not testing were explored (Q8). Finally, all participants were asked to propose potential solutions to increase the likelihood of CHE testing (Q9) (Table 1).

**Table 1:** A questionnaire on CHE management for physicians in cirrhosis care

Q1. Is CHE a significant problem?	Q7 is for those who answered “Yes” to Q6
Yes	Q7. Which medications do you use to treat CHE? (Select all that apply)
No	Lactulose
Q2. Should CHE be tested for?	Rifaximin
Yes	Branched-chain amino acids
No	Zinc
Q3. How often do you test CHE?	Levocarnitine
0%	Other
1–49%	Q8 is for those who answered 0% to Q3
50–80%	Q8. Why don’t you test for CHE? (Select all that apply)
> 80%	Adds time to clinic visit
Q4–6 are for those who answered > 0% to Q3	Difficult, expensive tests requiring trained personnel
Q4. Which tests for CHE are conducted in your practice? (Select all that apply)	Testing is not standardized in Japan
Neuropsychiatric test	Not sure if treatment is effective
Stroop test	Other
Animal naming test	Q9. What will increase your likelihood of testing for CHE? (Select all that apply)
Q5. Why do you test for CHE? (Select all that apply)	Simple tests that can be administered by clinic staff
CHE is associated with poor a poor quality of life	A testing system through a multidisciplinary team
CHE is associated with falls	Studies proving that CHE is associated with a poor quality of life
CHE is associated with motor vehicle accidents	Studies proving that CHE is associated with falls
CHE increases the risk of overt hepatic encephalopathy	Studies proving that CHE is associated with motor vehicle accidents
CHE is associated with a poor prognosis	Studies proving that CHE is associated with overt hepatic encephalopathy
Multidisciplinary team is working on CHE	Studies proving that CHE is associated with a poor prognosis
Others	Studies proving the effectiveness of CHE treatment
Q6. Do you treat CHE?	Others
Yes	
No	

*CHE* covert hepatic encephalopathy

### Data collection

The following baseline information was collected from physicians: age, gender, years of experience as a physician, type of institution, region of institution, and board certification. Participants were categorized into five age groups: < 30 years, 30–39 years, 40–49 years, 50–59 years, and ≥ 60 years and five ranges of years of experience: < 10 years, 10–19 years, 20–29 years, 30–39 years, and ≥ 40 years. Institutions were classified into three types: university hospital, general hospital, and other. Eight regions were defined: Hokkaido, Tohoku, Kanto, Chubu, Kansai, Chugoku, Shikoku, and Kyushu–Okinawa (Supplementary Fig. 1). Additionally, information on board certifications from the Japan Society of Hepatology (JSH), Japanese Society of Gastroenterology (JSGE), and Japanese Society of Internal Medicine (JSIM) was collected and analyzed.

### Statistical analysis

Baseline characteristics of participants were presented as numbers and percentages. Participants were divided into two groups based on CHE testing status: those who tested for CHE (Test group) and those who did not (No-test group). A chi-squared test was used to compare the two groups. Factors associated with the Test group were analyzed using a multivariable logistic regression model that included all baseline variables of the participants. Results were reported as odds ratios (ORs) with 95% confidence intervals (CIs). Participants with missing data were excluded from the analysis; therefore, no imputation was performed. All tests were two-sided, with a *p* value < 0.05 set as the threshold for statistical significance. All analyses were conducted using R software, version 4.4.1 (The R Foundation for Statistical Computing, Vienna, Austria).

## Results

### Baseline characteristics of physicians in cirrhosis care enrolled in the study

Of the 550 participants screened, 511 met the eligibility criteria and were included in the analysis (Supplementary Fig. 2). Baseline characteristics of the 511 participants are shown in Table 2. Of these participants, 445 (87.1%) were male. The majority were aged 40–49 years (36.8%), followed by 50–59 years (26.0%) and 30–39 years (25.8%). The majority had 10–19 years (36.0%) of clinical experience, followed by of 20–29 years (31.9%), and 30–39 years (17.0%). Regarding institutional affiliation, 54.2% of participants worked at university hospitals, and 44.0% worked at general hospitals. Regionally, the majority were from Chubu (28.2%), followed by Kanto (18.6%), Kansai (14.7%), Kyushu–Okinawa (14.1%), Tohoku (8.0%), Chugoku (6.5%), Hokkaido (5.1%), and Shikoku (4.9%). Regarding board certification, 82.8% of participants were certified by the JSH, 87.1% by the JSGE, and 69.1% by the JSIM (Table 2).

**Table 2:** Baseline characteristics of physicians in cirrhosis care divided by CHE testing status

Characteristic	Overall	Test group	No-test group	*p* value^*^
	(n = 511)	(n = 321)	(n = 190)	
Male gender	445 (87.1)	280 (87.2)	165 (86.8)	1.000
Age group				
< 30 years	14 (2.7)	9 (2.8)	5 (2.6)	0.838
30–39 years	132 (25.8)	80 (24.9)	52 (27.4)	
40–49 years	188 (36.8)	120 (37.4)	68 (35.8)	
50–59 years	133 (26.0)	87 (27.1)	46 (24.2)	
≥ 60 years	44 (8.6)	25 (7.8)	19 (10.0)	
Years of experience				
< 10 years	65 (12.7)	42 (13.1)	23 (12.1)	0.962
10–19 years	184 (36.0)	116 (36.1)	68 (35.8)	
20–29 years	163 (31.9)	104 (32.4)	59 (31.1)	
30–39 years	87 (17.0)	52 (16.2)	35 (18.4)	
≥ 40 years	12 (2.3)	7 (2.2)	5 (2.6)	
Institution				
University Hospital	277 (54.2)	194 (60.4)	83 (43.7)	< 0.001
General Hospital	225 (44.0)	125 (38.9)	100 (52.6)	
Others	9 (1.8)	2 (0.6)	7 (3.7)	
Region				
Hokkaido	26 (5.1)	16 (5.0)	10 (5.3)	0.376
Tohoku	41 (8.0)	28 (8.7)	13 (6.8)	
Kanto	95 (18.6)	62 (19.3)	33 (17.4)	
Chubu	144 (28.2)	91 (28.3)	53 (27.9)	
Kansai	75 (14.7)	42 (13.1)	33 (17.4)	
Chugoku	33 (6.5)	26 (8.1)	7 (3.7)	
Shikoku	25 (4.9)	16 (5.0)	9 (4.7)	
Kyushu–Okinawa	72 (14.1)	40 (12.5)	32 (16.8)	
Society certification				
JSH	423 (82.8)	276 (86.0)	147 (77.4)	0.018
JSGE	445 (87.1)	276 (86.0)	169 (88.9)	0.407
JSIM	353 (69.1)	233 (72.6)	120 (63.2)	0.033

Values are presented as numbers (percentages)

*CHE* covert hepatic encephalopathy, *JSGE* Japanese Society of Gastroenterology, *JSH *Japan Society of Hepatology, *JSIM* Japanese Society of Internal Medicine

^*^Statistical differences between the two groups were analyzed using the chi-square test

### Clinical perspectives and testing status for CHE

**Fig. 1:**
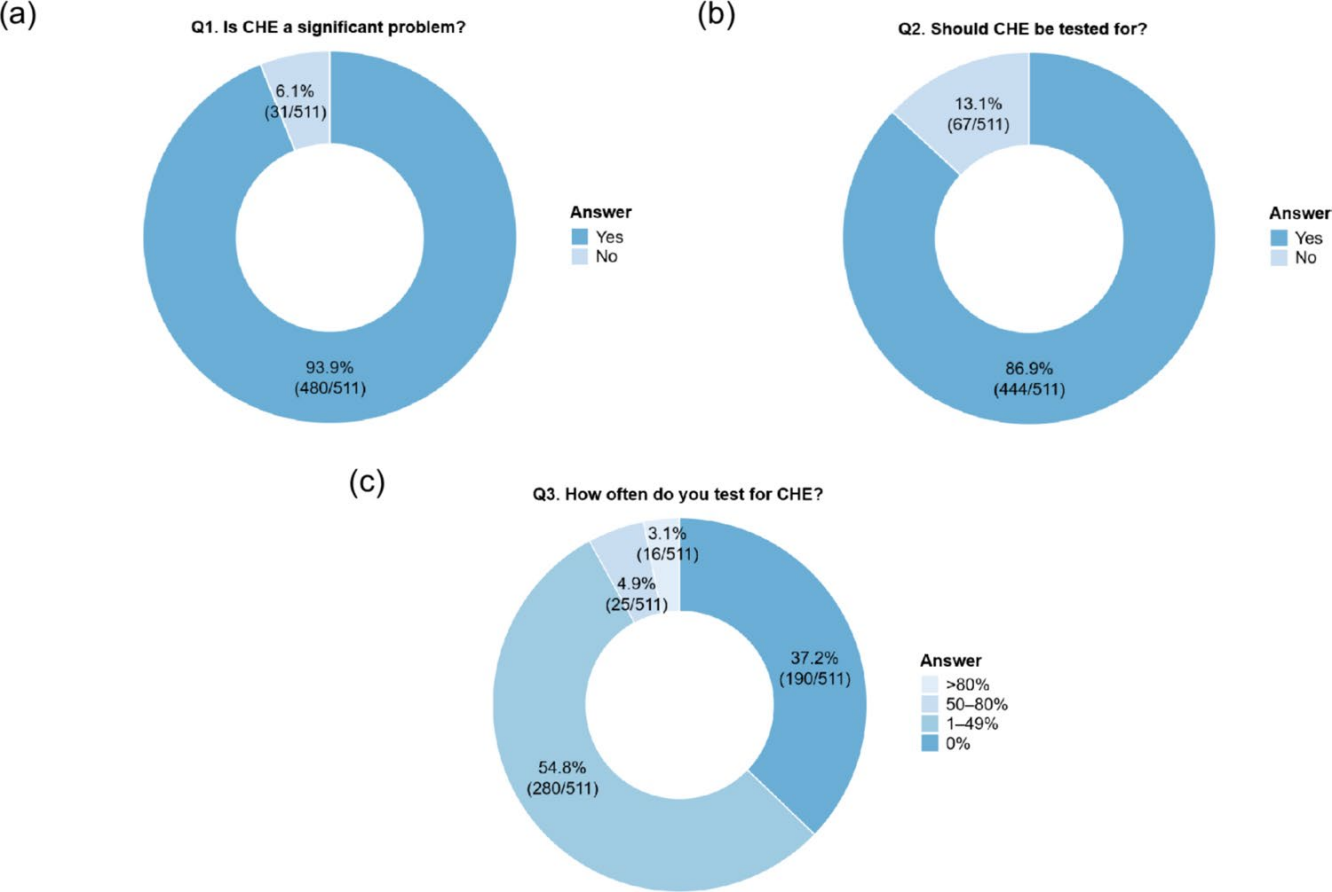
Questionnaire results for **a** “Q1: Is CHE a significant problem?”, **b** “Q2: Should CHE be tested for?”, and **c** “Q3: How often do you test for CHE?”. *CHE* covert hepatic encephalopathy

Among the 511 participants, 480 (93.9%) acknowledged that CHE is a significant issue in the management of cirrhosis (Fig. 1a). Similarly, 444 (86.9%) agreed that CHE should be tested for in patients with cirrhosis (Fig. 1b). However, only 280 (54.8%) reported conducting tests in 1–49% of their patients, whereas 190 (37.2%) stated that they do not perform CHE testing at all (Fig. 1c).

### Comparison between physicians who test CHE and those who do not

Among the 511 participants, the Test group comprised 321 (62.8%) and the No-test group comprised 190 (37.2%) (Supplementary Fig. 2). Physicians in the Test group were more likely to be affiliated with university hospitals (60.4 vs. 43.7%; *p* < 0.001) and to hold certifications from the JSH (86.0 vs. 77.4%; *p* = 0.018) and JSIM (72.6% vs. 63.2%; *p* = 0.033) than those in the No-test group. Conversely, no significant differences were observed between the groups in terms of gender, age, years of experience, or geographical region (Table 2).

### Determinants of CHE testing status

Multivariable logistic regression results assessing factors influencing the implementation of CHE testing are presented in Table 3. Among the baseline characteristics, working in general hospitals (OR: 0.46; 95% CI 0.31–0.69; *p* < 0.001) or other institutions (OR: 0.08; 95% CI 0.01–0.38; *p* = 0.003) was independently and negatively associated with the likelihood of CHE testing compared with working in university hospitals. Board certification by the JSH (OR: 5.82; 95% CI 2.56–14.42; *p* < 0.001) and the JSIM (OR: 1.61; 95% CI 1.02–2.54; *p* = 0.042) was positively associated with testing status, whereas board certification by the JSGE showed a negative association. Other factors, including age, gender, years of experience, and region, did not significantly influence CHE testing. Contrast analysis of No-test groups showed results with a comparable interpretation (Supplementary Table 1).

**Table 3:** Multivariable model for independent medical factors in testing for CHE

Characteristic	OR (95% CI)	*p* value^*^
Male gender	0.96 (0.52–1.72)	0.885
Age group		
<30 years^a^	1.00	
30–39 years	0.67 (0.17–2.46)	0.557
40–49 years	0.74 (0.16–3.23)	0.692
50–59 years	1.22 (0.24–6.03)	0.806
≥60 years	0.85 (0.13–5.26)	0.864
Years of experience		
<10 years^a^	1.00	
10–19 years	0.41 (0.13–1.23)	0.119
20–29 years	0.28 (0.07–1.01)	0.055
30–39 years	0.27 (0.06–1.16)	0.081
≥40 years	0.46 (0.06–3.59)	0.454
Institution		
University Hospital^a^	1.00	
General Hospital	0.46 (0.31–0.69)	<0.001
Others	0.08 (0.01–0.38)	0.003
Region		
Hokkaido^a^	1.00	
Tohoku	1.55 (0.50–4.72)	0.442
Kanto	1.15 (0.43–2.99)	0.775
Chubu	1.16 (0.45–2.90)	0.754
Kansai	0.83 (0.31–2.19)	0.714
Chugoku	2.89 (0.85–10.31)	0.093
Shikoku	1.09 (0.32–3.68)	0.895
Kyushu–Okinawa	0.83 (0.30–2.19)	0.707
Society certification		
JSH	5.82 (2.56–14.42)	<0.001
JSGE	0.36 (0.15–0.85)	0.022
JSIM	1.61 (1.02–2.54)	0.042

*CHE* covert hepatic encephalopathy, *JSGE* Japanese Society of Gastroenterology, *JSH* Japan Society of Hepatology, *JSIM* Japanese Society of Internal Medicine

^a^Reference group

^*^Multivariable analysis was performed using logistic regression

### Details of testing and treatment of CHE in physicians who test for CHE

**Fig. 2:**
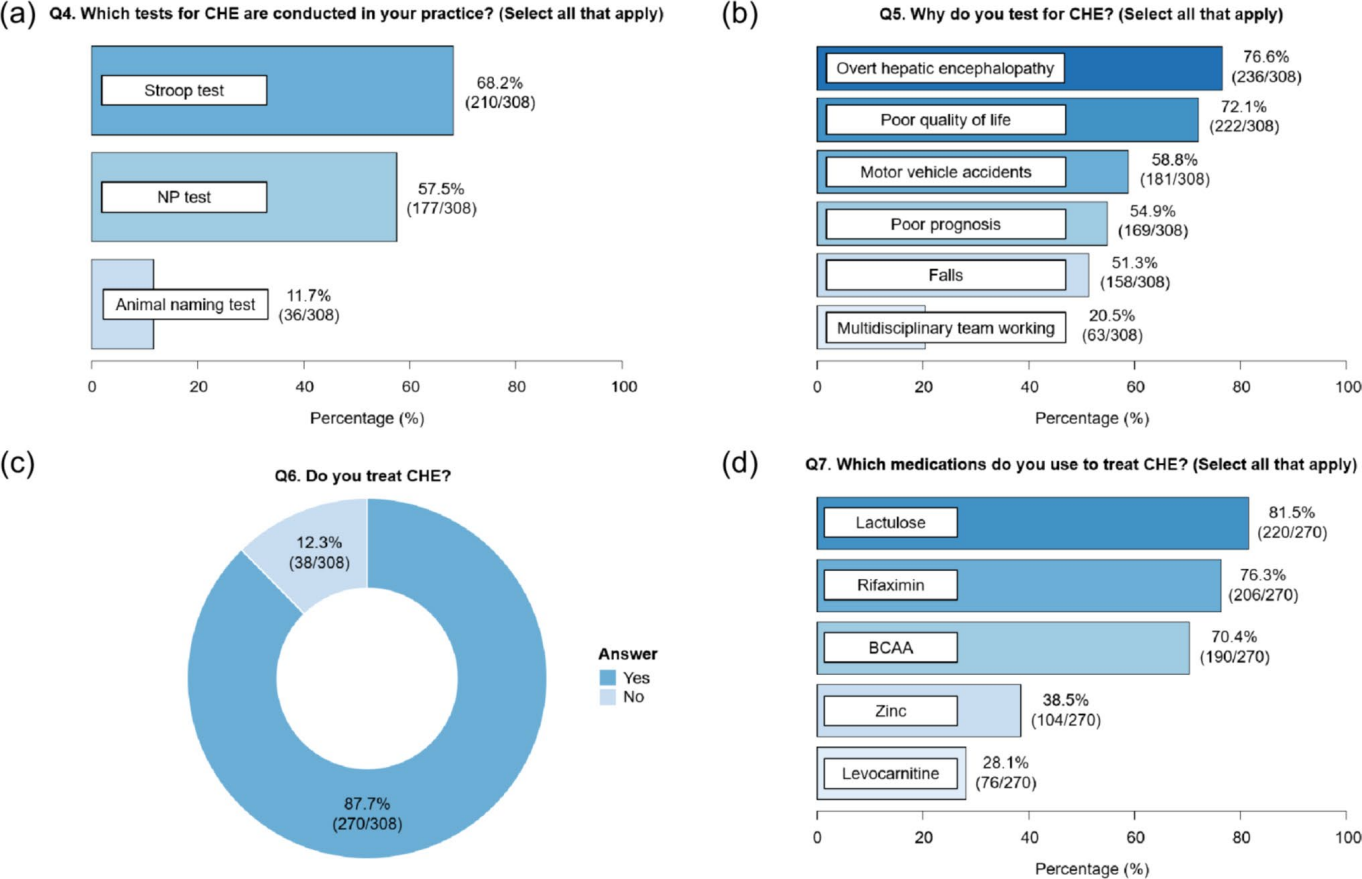
Questionnaire results for **a** “Q4: Which tests for CHE are conducted in your practice?”, **b** “Q5: Why do you test for CHE?”, **c** “Q6: Do you treat CHE?”, and **d** “Q7: Which medications do you use to treat CHE?”. *BCAA* branched-chain amino acids, *CHE* covert hepatic encephalopathy

Of the 321 participants who tested for CHE, 308 without missing data in Q4–Q8 were analyzed (Supplementary Fig. 2). In Japan, the Stroop test (68.2%) was the most commonly used method for CHE testing, followed by the neuropsychiatric (NP) test (57.5%) and the animal naming test (ANT) (11.7%) (Fig. 2a). Physicians cited several reasons for performing CHE tests, including an increased risk of OHE (75.6%), poor QOL (72.1%), motor vehicle accidents (58.8%), poor prognosis (54.9%), and falls (51.3%) (Fig. 2b). Among those who performed CHE tests, 270 (87.7%) indicated that they would initiate treatment if CHE was detected (Fig. 2c). Physicians who treat CHE were more likely to consider its impact on poor QOL compared to those who do not (Supplementary Table 2). The most commonly used therapies were lactulose (81.5%), followed by rifaximin (76.3%), branched-chain amino acids (BCAA) (70.4%), zinc (38.5%), and levocarnitine (28.1%) (Fig. 2d).

### Barriers and future perspectives for CHE testing

**Fig. 3:**
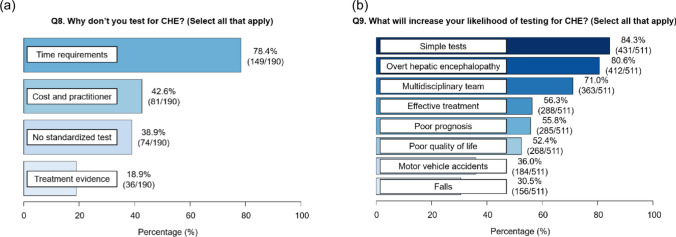
Questionnaire results for **a** “Q8: Why don’t you test for CHE?” and **b** “Q9: What will increase your likelihood of testing for CHE?”. *CHE* covert hepatic encephalopathy

Among the 190 participants who did not test for CHE, the primary barriers to CHE testing were time requirements (78.4%), cost and limited practitioner availability (42.6%), and the lack of standardized tests (38.9%) (Fig. 3a). Proposed solutions to enhance CHE testing included the development of simple tests (84.3%), evidence supporting the association between CHE and OHE (80.6%), the establishment of multidisciplinary teams (71.0%), and evidence of effective treatment (56.3%), poor prognosis (55.8%), and reduced QOL (52.4%) (Fig. 3b). Additionally, some physicians suggested that the development of biomarkers and health insurance coverage for testing costs could further encourage CHE testing.

## Discussion

CHE is a significant complication that impacts various clinical outcomes in patients with cirrhosis [1–5]. Therefore, routine screening for CHE is crucial to improving patient outcomes. However, discrepancies between available evidence and clinical practice limit the effective implementation of CHE management in cirrhosis care. In this nationwide survey, we evaluated physicians’ perspectives on CHE, current management practices including testing and treatment, barriers to CHE testing, and potential solutions to bridge the gap between evidence and clinical practice.

The first key finding highlights the perspectives of Japanese physicians on CHE and the rate of CHE testing among those involved in cirrhosis care. In a previous survey published in 2007, 84% of AASLD members acknowledged CHE as a significant problem, and 74% believed it should be tested for [10]. Compared with the AASLD report, the results of this Japanese survey indicate heightened awareness among physicians regarding the importance of CHE and the need for its testing (Supplementary Table 3). This is likely due to the accumulation of evidence since the previous survey, which has enhanced physicians' recognition of CHE in cirrhosis management and increased the clinical demand for CHE testing. However, the rate of CHE testing showed little difference between the AASLD report and the Japanese survey (0%/1–49%/50–80%/ > 80%: 38%/34%/14%/14% vs. 37%/55%/5%/3%) [10]. Notably, although the majority of physicians acknowledged CHE as a serious problem that required testing, 37% did not perform any CHE testing. In addition, physicians at university hospitals and those with board certification from the JSH or JSIM were more likely to actively perform CHE testing. These findings indicate that CHE testing is currently concentrated among specific medical institutions and physicians with a greater focus on CHE management. To enhance the uptake of CHE testing, it is essential to investigate this gap further and develop targeted strategies to address it.

The second key finding is the detailed characterization of CHE testing and treatment practices in Japan. The NP test, a gold-standard computerized test battery for diagnosing CHE in Japan [11, 12], demonstrates promising potential in predicting clinical outcomes [9, 13]. However, the complexity of the NP test, which requires more than 20 min to administer, limits its widespread adoption. The Stroop test is a point-of-care screening tool for CHE, offering promising accuracy in identifying CHE and predicting clinical outcomes [14, 15]. The findings of this study identified the Stroop test as the most commonly used screening tool for CHE in the Japanese population, which may reflect its shorter administration time compared with the NP test and recent efforts to validate its efficacy in diagnosing CHE and predicting clinical outcomes in this population [16–18]. The ANT can be completed in one minute and does not require any devices or well-trained practitioners [19]. However, the current survey revealed limited use of ANT among the Japanese population. Regarding the clinical relevance of CHE testing, Japanese physicians emphasized the evidence linking CHE to OHE and its association with poor QOL, whereas AASLD members primarily highlighted its association with QOL [10]. Notably, 87.7% of physicians who tested for CHE reported that they would initiate treatment upon diagnosis. Among Japanese physicians, the impact of CHE on QOL was a key factor in initiating treatment. Lactulose, rifaximin, and BCAA were the most commonly used treatments for managing CHE in Japan. Lactulose and rifaximin are validated as effective treatments for CHE [20], while the active use of BCAA is a notable practice among Japanese physicians. However, these findings should be interpreted with caution, as robust data confirming that the treatment of CHE reduces the incidence of OHE are lacking [3]. Treatment of CHE has been shown to improve CHE and QOL in patients with cirrhosis [20–22]. Accordingly, current guidelines recommend initiating CHE treatment and, if beneficial, it could also support the diagnosis of CHE [3]. Since evidence on CHE treatment and its impact on major outcomes remains limited, future studies should clarify its benefits and establish strategies for selecting appropriate medications.

The third key finding underscores the current limitations of CHE testing and proposes potential solutions to bridge management gaps. Among physicians who do not test for CHE, time requirements were identified as the primary barrier to CHE testing. Consequently, many physicians suggested the development of simple tests and the establishment of multidisciplinary teams to create an ideal environment for CHE testing. These findings align with the AASLD survey, which suggested that “simple tests that can be administered by clinic staff” could increase the likelihood of CHE testing [10]. The Stroop test and its shortened version offer a promising solution for CHE screening. The shortened test, which can be completed in under a minute, demonstrates comparable effectiveness to the standard version in identifying CHE and estimating the risk of progression to OHE [23–25]. In addition, the ANT, another simple test, has demonstrated potential for CHE screening [3]. Differences in national character, culture, and language may make CHE testing more challenging in Japan compared to other countries. Previous study has shown that the ANT can be conducted in Japanese patients with cirrhosis and the use of Oriental zodiac does not affect the performance in the ANT [26]. However, evidence on its effectiveness in identifying CHE in Japanese patients remains limited, considering the high education rate in Japan [26]. Furthermore, the social stigma surrounding neurological function tests in Japan may serve as a barrier to their implementation from the patient’s perspective. Therefore, future studies should establish simple tests in the Japanese population and assess barriers to testing from the patient’s perspective.

Another approach to increase the testing for CHE is management by a multidisciplinary team. Liver functional reserve, nutritional status, sarcopenia, medications, and CHE are recognized as risk factors for poor outcomes in patients with cirrhosis [27–29]. Given the strong interplay among these risk factors, establishing a multidisciplinary team to comprehensively assess, treat, and manage them could enhance the quality of cirrhosis care and address gaps in CHE management [30, 31]. Nurses play a central role in monitoring cognitive function and educating patients to improve their adherence to treatment [32]. Dietitians provide individualized nutritional counseling, including nutritional assessment and optimization of nutritional interventions, to improve nutritional status, which impacts CHE [33]. Conducting CHE testing during nutritional counseling may be an effective strategy for detecting CHE. Physical therapists develop exercise programs to improve sarcopenia and frailty, both of which increase the risk of CHE and falls [34, 35]. Pharmacists conduct medication reviews to recommend appropriate therapies and minimize the use of inappropriate treatments in the management of CHE [29]. Finally, sharing information and providing mutual suggestions can help establish a multidisciplinary team of specialists who actively engage in the screening and management of CHE, thereby facilitating early detection, optimizing treatment quality, and improving outcomes for patients with cirrhosis.

In Japan, CHE testing is not covered by public insurance and, the health insurance coverage for testing costs and the development of simple biomarkers to identify high-risk populations are important for increasing the likelihood of CHE testing. Due to the concentration of the population in urban areas, the implementation of CHE testing can be influenced by the uneven distribution of specialists and limited availability of tools across institutions. With the growing proportion of the aging population, the early neurological changes in CHE are becoming increasingly difficult to distinguish from other conditions such as dementia [36]. Furthermore, the structure of the medical system, where decisions to perform tests are largely guided by physicians’ knowledge and expertise, can influence the status of testing. Therefore, further research is necessary to address and explore these issues.

This study has several limitations. First, the self-reported nature of the questionnaire survey may not accurately represent actual clinical records or practices. Second, a Japanese survey may limit the generalizability of the results to other regions. Therefore, further studies involving international populations are necessary to assess global trends and limitations in CHE management and to validate the findings of our study. Nevertheless, the strengths of our study should be highlighted, including its nationwide scope, adequate sample size, and findings that are strongly corroborated by the previous survey [10].

In conclusion, physicians involved in cirrhosis care recognize CHE as a significant problem that should be tested for in patients with cirrhosis. However, more than one-third of physicians do not test for CHE, and its implementation remains limited by institutional factors and physicians’ specialties. Time requirements are the primary barrier to testing, and, therefore, the development of simple tests and the establishment of multidisciplinary teams are essential to enhance CHE management in cirrhosis care.

## Supplementary Information

Below is the link to the electronic supplementary material.

Supplementary file1 (DOCX 19 KB)

Supplementary file2 (DOCX 18 KB)

Supplementary file3 (DOCX 20 KB)

Supplementary file4 (DOCX 123 KB)

Supplementary file5 (DOCX 226 KB)
