# Supplementary material for: Current management and future perspectives of covert hepatic encephalopathy in Japan: a nationwide survey
Source: J Gastroenterol. 2025 Mar 7;60(7):866–76. doi: 10.1007/s00535-025-02232-0 (PMC12177000; doi:10.1007/s00535-025-02232-0)
Supplement: Supplementary file 1 — Supplementary file1 (DOCX 19 KB) [file 535_2025_2232_MOESM1_ESM.docx]

| Characteristic | OR (95% CI) | *p*-value^*^ |
| --- | --- | --- |
| Male gender | 1.04 (0.58–1.91) | 0.885 |
| Age group |  |  |
| <30 years^a^ | 1.00 |  |
| 30–39 years | 1.48 (0.41–5.85) | 0.557 |
| 40–49 years | 1.35 (0.31–6.30) | 0.692 |
| 50–59 years | 0.82 (0.17–4.25) | 0.806 |
| ≥60 years | 1.17 (0.19–7.60) | 0.864 |
| Years of experience |  |  |
| <10 years^a^ | 1.00 |  |
| 10–19 years | 2.45 (0.81–7.83) | 0.119 |
| 20–29 years | 3.56 (0.99–13.44) | 0.055 |
| 30–39 years | 3.76 (0.86–17.03) | 0.081 |
| ≥40 years | 2.15 (0.28–15.94) | 0.454 |
| Institution |  |  |
| University Hospital^a^ | 1.00 |  |
| General Hospital | 2.17 (1.45–3.26) | <0.001 |
| Others | 11.90 (2.64–84.30) | 0.003 |
| Region |  |  |
| Hokkaido^a^ | 1.00 |  |
| Tohoku | 0.65 (0.21–1.98) | 0.442 |
| Kanto | 0.87 (0.33–2.34) | 0.775 |
| Chubu | 0.86 (0.35–2.24) | 0.754 |
| Kansai | 1.20 (0.46–3.26) | 0.714 |
| Chugoku | 0.35 (0.1–1.18) | 0.093 |
| Shikoku | 0.92 (0.27–3.1) | 0.895 |
| Kyushu–Okinawa | 1.21 (0.46–3.31) | 0.707 |
| Society certification |  |  |
| JSH | 0.17 (0.07–0.39) | <0.001 |
| JSGE | 2.77 (1.18–6.82) | 0.022 |
| JSIM | 0.62 (0.39–0.98) | 0.042 |

**Supplementary Table 1.** Multivariable model for independent medical factors in non-testing for CHE

^*^Multivariable analysis was performed using logistic regression.

^a^Reference group

Abbreviations: CHE, covert hepatic encephalopathy; JSGE, Japanese Society of Gastroenterology; JSH, Japan Society of Hepatology; JSIM, Japanese Society of Internal Medicine
