# Supplementary material for: Current management and future perspectives of covert hepatic encephalopathy in Japan: a nationwide survey
Source: J Gastroenterol. 2025 Mar 7;60(7):866–76. doi: 10.1007/s00535-025-02232-0 (PMC12177000; doi:10.1007/s00535-025-02232-0)
Supplement: Supplementary file 2 — Supplementary file2 (DOCX 18 KB) [file 535_2025_2232_MOESM2_ESM.docx]

**Supplementary Table 2.** Comparison of reasons for testing CHE between physicians who treat CHE and those who do not

|  | Q6. Do you treat CHE? | |  |
| --- | --- | --- | --- |
|  | Yes | No | *p*-value* |
| Q5. Why do you test for CHE? | (n = 270) | (n = 38) |  |
| Poor quality of life, n (%) | 203 (75.2) | 19 (50.0) | 0.002 |
| Falls, n (%) | 143 (53.0) | 15 (39.5) | 0.166 |
| Motor vehicle accidents, n (%) | 163 (60.4) | 18 (47.4) | 0.178 |
| Overt hepatic encephalopathy, n (%) | 207 (76.7) | 29 (76.3) | 1.000 |
| Poor prognosis, n (%) | 154 (57.0) | 15 (39.5) | 0.062 |
| Multidisciplinary team working, n (%) | 60 (22.2) | 3 (7.9) | 0.066 |

Values are presented as numbers (percentages).

*Statistical differences between the two groups were analyzed using the chi-square test.

Abbreviations: CHE, covert hepatic encephalopathy
