# Supplementary material for: Current management and future perspectives of covert hepatic encephalopathy in Japan: a nationwide survey
Source: J Gastroenterol. 2025 Mar 7;60(7):866–76. doi: 10.1007/s00535-025-02232-0 (PMC12177000; doi:10.1007/s00535-025-02232-0)
Supplement: Supplementary file 3 — Supplementary file3 (DOCX 20 KB) [file 535_2025_2232_MOESM3_ESM.docx]

**Supplementary Table 3.** Comparison of the Japanese and American surveys

| Questionnaire items | Japanese survey | American survey^a^ |
| --- | --- | --- |
| Q1. Is CHE a significant problem? |  |  |
| ・Yes | 93.9% | 84% |
| ・No | 6.1% | 16% |
| Q2. Should CHE be tested for? |  |  |
| ・Yes | 86.9% | 74% |
| ・No | 13.1% | 26% |
| Q3. How often do you test CHE? |  |  |
| ・0% | 37.2% | 38% |
| ・1–49% | 54.8% | 34% |
| ・50–80% | 4.9% | 14% |
| ・>80% | 3.1% | 14% |
| Q4–6 are for those who answered >0% to Q3. |  |  |
| Q4. Which tests for CHE are conducted in your practice? (Select all that apply) |  |  |
| ・Neuropsychiatric test | 68.2% | NA |
| ・Stroop test | 57.5% | NA |
| ・Animal naming test | 11.7% | NA |
| Q5. Why do you test for CHE? (Select all that apply) |  |  |
| ・CHE is associated with poor a poor quality of life | 72.1% | 86% |
| ・CHE is associated with falls | 51.3% | NA |
| ・CHE is associated with motor vehicle accidents | 58.8% | 74% |
| ・CHE increases the risk of overt hepatic encephalopathy | 76.6% | 52% |
| ・CHE is associated with a poor prognosis | 54.9% | 31% |
| ・Multidisciplinary team is working on CHE | NA | NA |
| Q6. Do you treat CHE? |  |  |
| ・Yes | 87.7% | NA |
| ・No | 12.3% | NA |
| Q7 is for those who answered "Yes" to Q6. |  |  |
| Q7. Which medications do you use to treat CHE? (Select all that apply) |  |  |
| ・Lactulose | 81.5% | NA |
| ・Rifaximin | 76.3% | NA |
| ・Branched-chain amino acids | 70.4% | NA |
| ・Zinc | 38.5% | NA |
| ・Levocarnitine | 28.1% | NA |
| Q8 is for those who answered 0% to Q3. |  |  |
| Q8. Why don’t you test for CHE? (Select all that apply) |  |  |
| ・Adds time to clinic visit | 78.4% | 85% |
| ・Difficult, expensive tests requiring trained personnel | 42.6% | 75% |
| ・Testing is not standardized | 38.9% | 69% |
| ・Not sure if treatment is effective | 18.9% | 42% |
| Q9. What will increase your likelihood of testing for CHE? (Select all that apply) |  |  |
| ・Simple tests that can be administered by clinic staff | 84.3% | 85% |
| ・A testing system through a multidisciplinary team | 80.6% | NA |
| ・Studies proving that CHE is associated with a poor quality of life | 52.6% | 42% |
| ・Studies proving that CHE is associated with falls | 30.5% | NA |
| ・Studies proving that CHE is associated with motor vehicle accidents | 36.0% | 49% |
| ・Studies proving that CHE is associated with overt hepatic encephalopathy | 80.6% | 54% |
| ・Studies proving that CHE is associated with a poor prognosis | 55.8% | 42% |
| ・Studies proving the effectiveness of CHE treatment | 56.3% | NA |

^a^Data demonstrated in Reference 10

Abbreviations: CHE, covert hepatic encephalopathy; NA, not available
