# Supplementary figures and images for: Current management and future perspectives of covert hepatic encephalopathy in Japan: a nationwide survey
Source: J Gastroenterol. 2025 Mar 7;60(7):866–76. doi: 10.1007/s00535-025-02232-0 (PMC12177000; doi:10.1007/s00535-025-02232-0)

**Supplementary Figure 1.** Regional divisions of Japan in the study


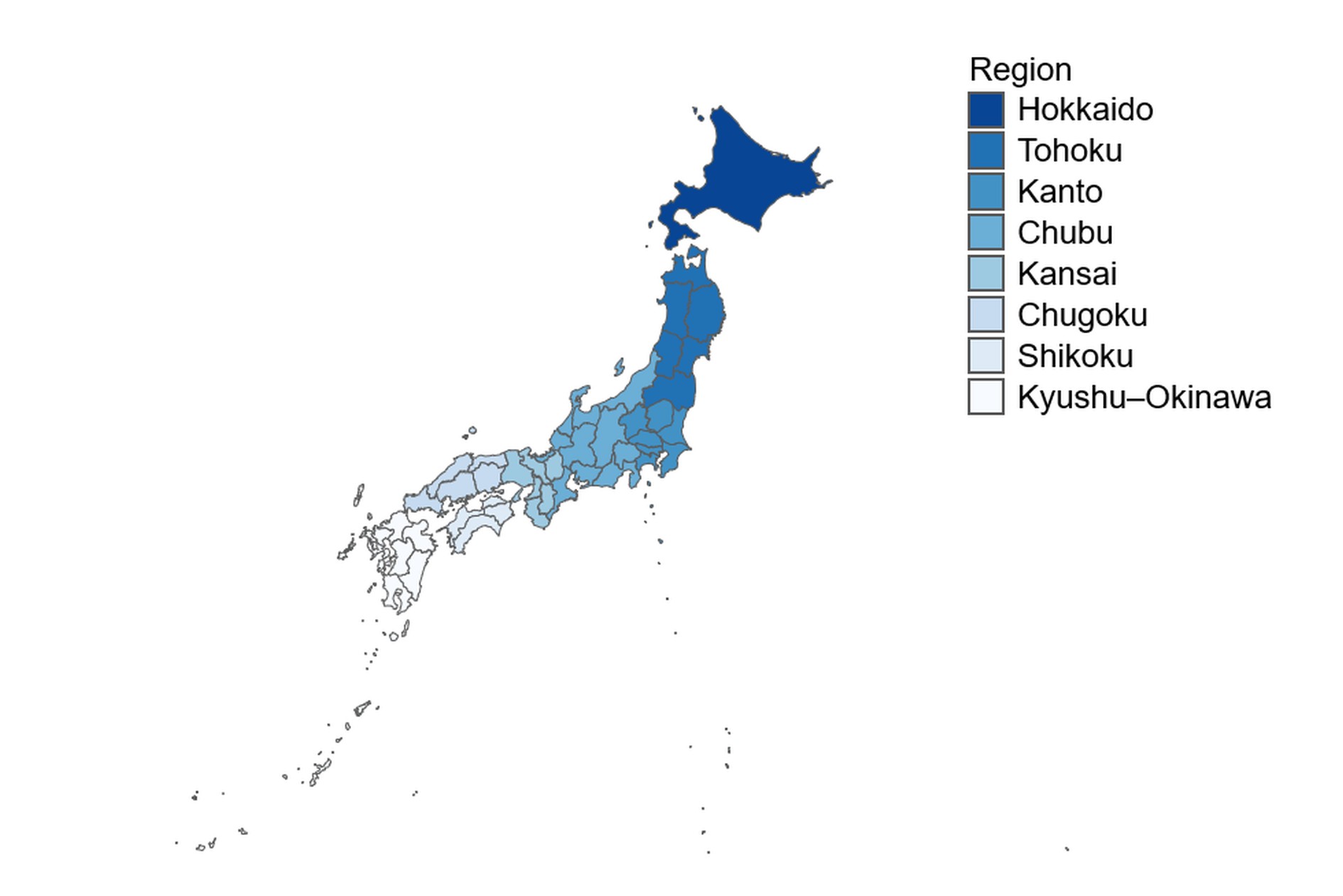

Supplement: Supplementary file 4 — Supplementary file4 (DOCX 123 KB) [file 535_2025_2232_MOESM4_ESM.docx]

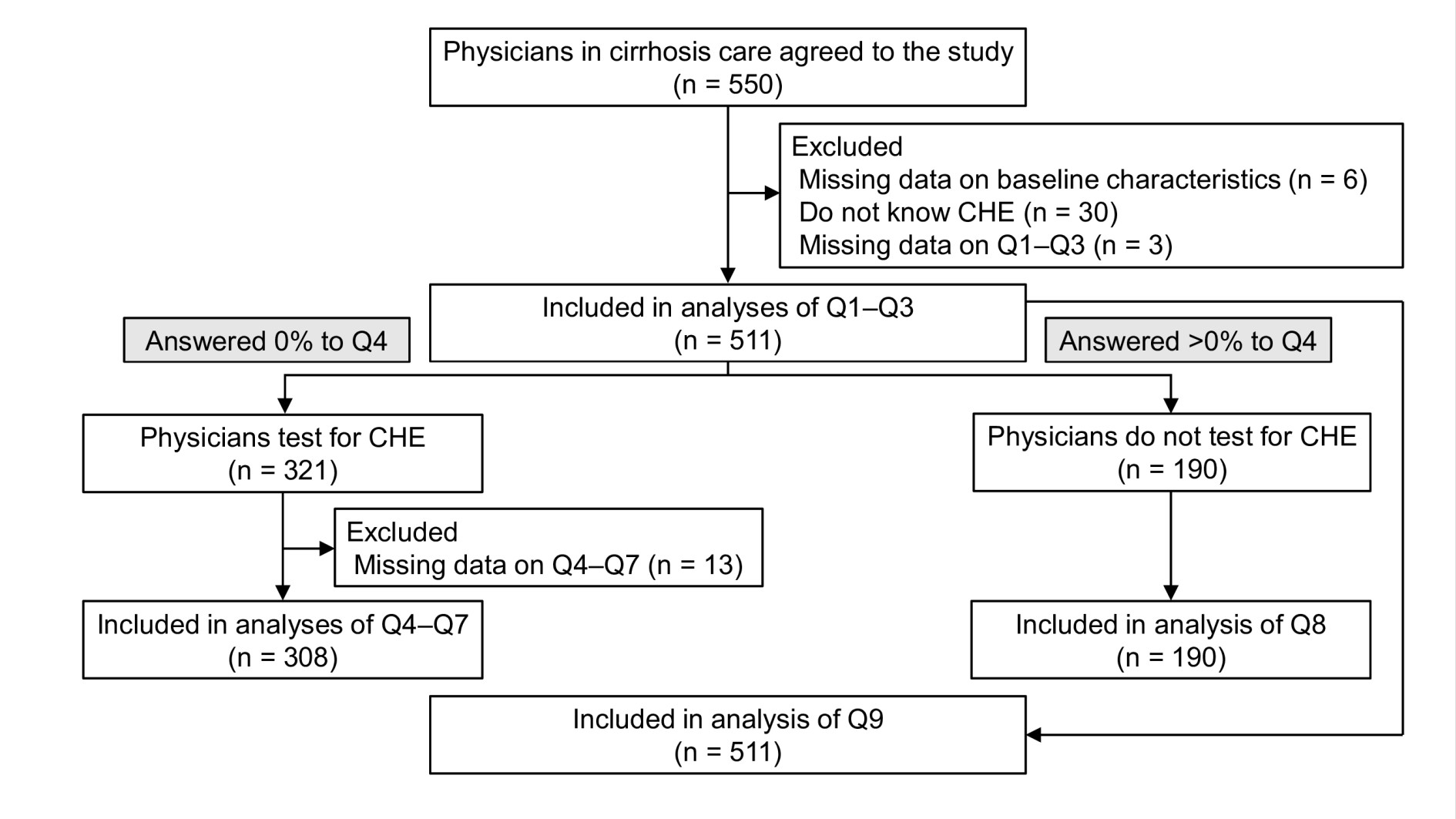
**Supplementary Figure 2.** Flow diagram of the study

Supplement: Supplementary file 5 — Supplementary file5 (DOCX 226 KB) [file 535_2025_2232_MOESM5_ESM.docx]
